# Sensors and Biosensors for C-Reactive Protein, Temperature and pH, and Their Applications for Monitoring Wound Healing: A Review

**DOI:** 10.3390/s17122952

**Published:** 2017-12-19

**Authors:** Pietro Salvo, Valentina Dini, Arno Kirchhain, Agata Janowska, Teresa Oranges, Andrea Chiricozzi, Tommaso Lomonaco, Fabio Di Francesco, Marco Romanelli

**Affiliations:** 1 Institute of Clinical Physiology, National Council of Research (IFC-CNR), Via Moruzzi 1, 56124 Pisa, Italy; 2 Department of Chemistry and Industrial Chemistry, University of Pisa, Via Moruzzi 13, 56124 Pisa, Italy; a.kirchhain@studenti.unipi.it (A.K.); tommaso.lomonaco@unipi.it (T.L.); fabio.difrancesco@unipi.it (F.D.F.); 3 Department of Dermatology, University of Pisa, Via Roma 67, 56126 Pisa, Italy; valentina.dini@unipi.it (V.D.); dottoressajanowska@gmail.com (A.J.); teresa.oranges@gmail.com (T.O.); chiricozziandrea@gmail.com (A.C.); m.romanelli@med.unipi.it (M.R.)

**Keywords:** C-reactive protein, temperature, pH, sensors, biosensors, wound healing

## Abstract

Wound assessment is usually performed in hospitals or specialized labs. However, since patients spend most of their time at home, a remote real time wound monitoring would help providing a better care and improving the healing rate. This review describes the advances in sensors and biosensors for monitoring the concentration of C-reactive protein (CRP), temperature and pH in wounds. These three parameters can be used as qualitative biomarkers to assess the wound status and the effectiveness of therapy. CRP biosensors can be classified in: (a) field effect transistors, (b) optical immunosensors based on surface plasmon resonance, total internal reflection, fluorescence and chemiluminescence, (c) electrochemical sensors based on potentiometry, amperometry, and electrochemical impedance, and (d) piezoresistive sensors, such as quartz crystal microbalances and microcantilevers. The last section reports the most recent developments for wearable non-invasive temperature and pH sensors suitable for wound monitoring.

## 1. Introduction

In normal physiological conditions, wound healing is a biological process that consists of four phases: hemostasis, inflammation, proliferation and tissue re-modeling [1]. Inflammation is an adaptive body reaction to physiological and pathological threats, such as traumatic, infectious, post-ischemic, toxic or autoimmune injuries [2,3]. For example, inflammation is associated with cardiovascular diseases, cancer, metabolic disorders, stress, diabetes, skin and respiratory diseases [3,4,5,6,7,8,9]. In wound healing, tissue repair starts during inflammation with the production of neutrophils, macrophages and lymphocytes that stimulate angiogenesis and attack external agents such as bacteria and viruses. During inflammation, the injured tissues produce exudate, i.e., a fluid rich in electrolytes, creatinine, fibrinogen, matrix metalloproteinases (MMPs), and proteins such as the tumor necrosis factor alpha (TNF-α), neutrophil gelatinase-associated lipocalin (NGAL), and the C-reactive protein (CRP) [1,10,11,12]. Human CRP is an annular calcium-dependent ligand-binding plasma protein composed of five identical non-glycosylated polypeptide sub-units with cyclic pentameric symmetry [13]. CRP is mainly synthesized in the liver upon an acute inflammatory stimulus, but some evidences indicate the production of CRP also in the kidneys and atherosclerotic tissues [14]. During the acute-phase response of inflammation, the CRP concentration in blood abruptly increases from about 0.8 mg/L to 600–1000 mg/L, reaching the peak value after about 48 h [14,15]. The half-life of CRP is about 19 h and the concentration in blood rapidly returns to basal values when the stimulus for the increased production ceases [13]. In the clinical setting, the most common CRP detection methods include immunonephelometric and immunoturbidimetric assays using a single polyclonal antibody, but there is also a wide diffusion of enzyme-linked immunosorbent assays (ELISA). However, these methods are time consuming and need specialized personnel [16,17].

Although there is no clear correlation between the CRP concentration and disease severity, high values of CRP reflect inflammation and/or tissue damage more precisely than other factors such as plasma viscosity and erythrocyte sedimentation rate [13]. CRP was suspected to promote tissue repair by enhancing the opsonization of microorganisms and the phagocytosis of necrotic and apoptotic cells, thus improving wound healing and reducing wound infection [18,19,20]. Furthermore, CRP was also associated with the regulation of clotting and the release of potentially destructive enzymes [21,22,23]. In 1999, Trengove et al. suggested that chronic wounds fail to heal due to a persistent inflammatory condition after finding decreased CRP levels in exudate samples from a small number of patients upon the improvement of the wound status [24]. A recent study confirmed the connection between CRP and wound healing by showing increased CRP blood levels in forty-one patients with chronic venous leg ulcers, when compared with the levels in an ulcer-free control group. Within the patients’ group, eight subjects with wound complications (e.g., infection) had higher CRP levels (average ~35 mg/L) than the subjects without complications (average ~9 mg/L). A concentration above 15 mg/mL was assumed indicative of wound inflammation [25]. The same study observed decreasing CRP concentrations over time in case of healing. Wound healing and CRP level were also associated in a study on burns, where acute inflammation and difficulty in wound healing corresponded to high CRP levels [26]. Kingsley et al. investigated whether the CRP level could be used as a marker of wound infection [27]. They grouped sixty-four patients in four categories with different severity of wound infection (colonization, critical colonization, local infection and spreading infection) and found high CRP blood levels in patients who belonged to the spreading infection group, but the discrimination between the other groups was not possible.

If the CRP level can be used as a non-specific indicator of wound healing, other parameters such as wound temperature and pH can support the evaluation of wound status. The pH measurement for wound assessment is described in [28,29], whereas the importance of wound temperature in the healing process was firstly reported in “De Medicina” from Aulus Cornelius Celsus, an ancient Roman encyclopaedist, and continued till recent times with a systematic study published in 1984 [30]. In 2013, a review paper included pH and temperature among the predictors of wound healing outcome [31]. Publications on the relation of wound healing with pH and temperature are quite varied in number of enrolled patients (from 7 to 362), aetiology (burns, pressure ulcers, surgical ulcers, vascular ulcers and diabetic foot ulcers), methods for pH measurement (pH meters, glass surface electrodes, pH indicator strips and litmus paper strips) and temperature measurement (non-contact infrared thermography, contact thermography and infrared thermometry) [32].

The pH of intact healthy skin is acidic and ranges from 4 to 7, whereas wounds with pH values greater than 7 fail to heal [1,26]. Romanelli et al. used a wound cleanser in patients with leg ulcers and correlated the decrease of the mean pH level to the reduction of the mean wound size [33]. Ono et al. described a statistically significant correlation between low pH and wound healing, without infection [34]. Shukla et al. observed a correlation between decreased pH levels and improved wound bed features and reported that the proliferation of the fibroblasts, vessels and keratinocytes, and the control of bacteria were favoured in an acidic environment [28]. Therefore, minimal variations in the pH value may alter the wound healing process [35].

Studies investigating the role of temperature in wound healing are largely heterogeneous due to the presence of factors such as inflammation, immune response, bacterial burden and high tissue metabolism. The neutrophils, fibroblasts, and epithelial cells activities decrease at temperatures lower than 33 °C and impair wound healing, whereas temperatures in the range 36–38 °C seem to promote the reduction of ulcer area [36,37]. Several works suggested that temperature monitoring could prevent ulceration [38,39,40]. In another study, the wound bed score of chronic wounds (range 0–16, the higher the better) improved for wound bed temperatures between 33 and 35 °C [41]. A temperature increase can also predict the onset of an infection [42]. Armstrong et al. studied the progression of wound healing in 332 diabetic foot patients using infrared thermometers and observed that the patients with a temperature difference at baseline between the infected and the healthy feet ≥ about 5.5 °C were characterized by non-healing and acute wound, whereas differences ≤ 5.5 °C were associated with the wounds in the healing phase [43]. Robicsek et al. found that peristernal skin temperatures >35 °C were associated with a high risk of sternal wound infection [30]. An observational non-randomised cohort study of thermographic patterns of surgical sites, which healed in uncomplicated orthopaedic surgery, showed similar results [44]. Hazenberg et al. found that the combination of temperature evaluation and photographic assessment increase the sensitivity (>60%) and the specificity (>79%) of the diagnosis of wound infection [45].

Literature reports that some CRP functions are regulated by pH, e.g., at acidic pH the native pentameric form of CRP transforms into another pentameric form capable of binding to other molecules such as factor H [46], whereas CRP assumes a compact monomeric form at basic pH [47]. Because of the correlation among CRP, pH, and temperature, these three parameters can provide supporting information to assess wound healing. This review describes sensors and biosensors that can be used to monitor the CRP, pH and temperature levels in wounds in order to provide a better care to patients and promote wound healing.

## 2. Sensors for C-Reactive Protein

The most common method to measure the CRP level is based on immunoassays, e.g., enzyme linked immunosorbent assay (ELISA), that need to be performed in specialized labs with time consuming procedures and expensive kits. ELISA is a well-established method, but it is affected by non-specific bindings. This review will focus on those technologies that can potentially improve the performances of current commercial methods.

### 2.1. Field Effect Transistors

Field Effect Transistors (FETs) are widely investigated to detect biomolecules since they are suitable for label-free sensors, low cost mass production and miniaturization, and are easy to integrate in other devices. The detection of biomolecules by FETs relies on the change of the channel conductivity between source and drain upon the interaction of target molecules with the transistor surface. The detection of CRP is commonly accomplished by immobilizing CRP antibodies (anti-CRP) onto the FET surface, typically on the gate oxide. CRP brings negative charges when binding to anti-CRP and alters the drain-source current (I_DS_). This change can be associated with the CRP concentration after a calibration [48].

In 2007, Sohn et al. published one of the first attempts to detect CRP levels by FET technology using anti-CRP anchored to the gate [49]. The authors adopted an extended gate configuration (EGFET), i.e., the sensing layer was physically external to the FET, but wired to its gate. This EGFET was tested in phosphate buffered saline (PBS) solution and showed the possibility to detect CRP by FETs in the range 3–10 µg/mL. Lyu et al. followed the same configuration, but their EGFET was only tested at a single CRP concentration [50].

In 2008, an *n*-channel FET was proposed to detect the CRP concentration in the range 3–20 µg/mL in PBS [51]. This FET used a gate insulator made of SiO_2_ and Si_3_N_4_, and an Au/NiCr gate. The authors used an Ion Selective FET (ISFET) configuration with a reference Ag/AgCl electrode. The functionalization of the gate by cysteine-tagged protein G helped the formation of a monoclonal anti-CRP layer, whereas bovine serum albumin (BSA) was used to avoid non-specific bindings.

Lee et al. fabricated freestanding silicon nanowires on a silicon substrate (SiNW-FET) [52]. Nanowires were chosen because the depletion/accumulation of electrical carriers is in the bulk rather than on the surface of the nanowires, thus leading to a greater sensitivity than in planar FETs, where the effect of the bound charges is lower. The SiNWs surfaces were functionalized with an aldehyde-terminated monolayer where anti-CRP were immobilized. The SiNW-FET was tested in a PBS solution with a salt concentration of 1.37 mM and CRP concentrations of 1 fM, 1pM and 1 nM were detected. Sensitivity was not reported. The same group used the SiNW-FETs to perform a clinical trial and detect CRP levels in 83 gastric cancer patients [53]. An aldehyde-terminated monolayer was formed onto the SiNWs before anchoring the anti-CRP. The selectivity was confirmed by testing the SiNW-FET in PBS with the prostate-specific antigen (PSA) and the tumor marker carcinoembryonic antigen (CEA). The transistor did not respond to CEA and PSA injections, but only to CRP (1 ng/mL). As proof-of-concept, six SiNW-FETs were used to detect CRP concentrations spanning from 3.2 to 10.4 µg/mL in the sera of six patients. However, because of measurement noise, this sensor could hardly discriminate between different concentrations. Lee et al. improved this SiNW-FET by using sol-gel materials to immobilize the anti-CRP, which allowed the antibodies’ activity to be maintained for months [54]. The test in human serum showed a linear sensor response to CRP in the range 0.12–10 ng/mL, but only one test was reported.

**Figure 1:**
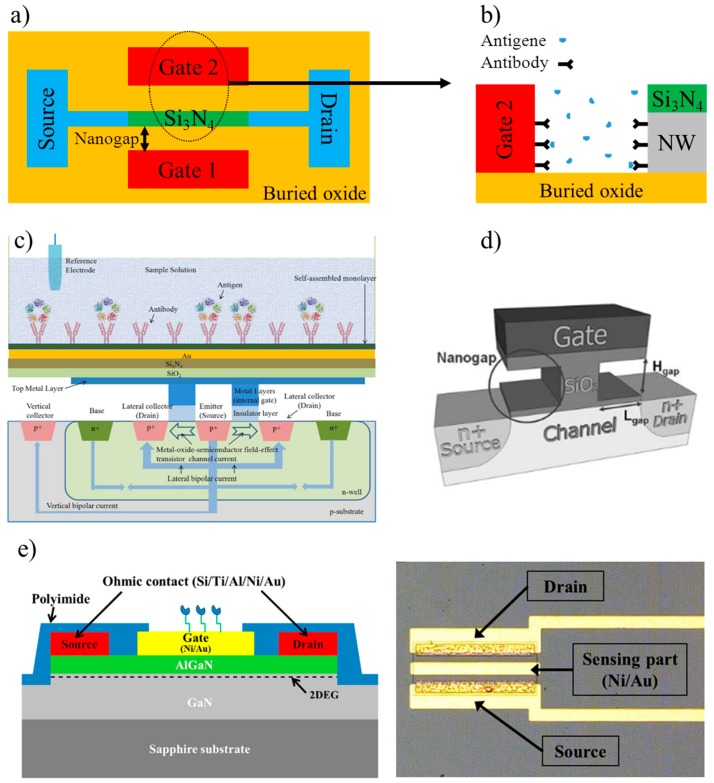
(**a**) Schematization of the nanogap-embedded NG-SiNW-FET (top view) and (**b**) detail of the use as immunosensor for CRP (Adapted from [55]); (**c**) schematization of the hybrid MOSFET-BJT. The BJT base current could be used for tuning the sensitivity for CRP detection (Adapted with permission from [57]); (**d**) schematic view of the nanogap-embedded FET with the gate dielectric made of air and SiO_2_ (Adapted with permission from [58]); (**e**) schematic view of a HEMT for detecting CRP. 2DEG is the two dimensional electron gas at the interface modulated by CRP binding ([62], licensed under CC BY 4.0).

Ahn et al. proposed a nanogap-embedded nanowire FET (NG-SiNW-FET) to detect CRP concentration in a PBS solution at pH 7.4 [55]. Figure 1a,b shows the NG-SiNW-FET and the location of the anti-CRP. The NG-SiNW-FET was fabricated on a silicon-on-insulator (SOI) substrate, whereas the gate insulator was Si_3_N_4_. The thickness of the nanogap was 30 nm, whereas the nanowire was 90 nm wide and 1 µm long. The threshold voltage of the NG-SiNW-FET had a shift of about 1.3 V when 100 μg/mL were cast on the device; however, no repeatability or sensitivity tests were reported. An improved SiNW-FET was described by Kwon et al. [56]. First, the SiNW surface was plasma-treated with oxygen to attach functional –OH groups. A 5% solution of 3-aminopropyltriethoxysilane (APTES) was then added to form amine groups. The SiNW was then exposed to 25 wt % glutaraldehyde with sodium cyanoborohydride (NaBH_3_CN). This process led to the formation of aldehyde groups that bound anti-CRP (100 µg/mL). Ethanolamine was eventually added to block non-specific binding. The width of the nanowire was 221 nm, which was recognized as the best compromise between a too narrow (i.e., low current level and more noise) and a too wide (i.e., high current level and less sensitivity) nanowire. The SiNW-FET was tested in PBS and the relative change of the nanowire conductance ranged from 39% with 10 µg/mL of CRP to 16% with 100 ng/mL.

In 2011, a hybrid metal-oxide FET (MOSFET) – bipolar junction transistor (BJT) was proposed to improve the limit of detection [57]. Figure 1c shows the schematic structure of the MOSFET-BJT, where the floating gate of the gated lateral BJT was first exposed to a solution of 11-mercaptoundecanoic acid (1 mmol/L in ethanol solution) and then to a solution containing 50 mmol/L N-Hydroxysuccinimide and 50 mmol/L N-(3-Dimethylaminopropyl)-N′-ethylcarbodiimide hydrochloride. The anti-CRP (50 µg/mL in PBS) were eventually anchored to the surface. The reference electrode was Ag/AgCl. The gated lateral BJT showed a lower concentration limit of detection (LOD) of 1 pmol/L (the response was evaluated as the variations of the emitter current). Interestingly, when used in the hybrid mode, the base current could be used for tuning the MOSFET channel current, i.e., the emitter current and the sensitivity for CRP detection. The selectivity was verified with the troponin T antigen, which induced no changes in the emitter current.

Kim et al. presented a FET with a 50 nm high and 450 nm long nanogap in the SiO_2_ gate oxide (Figure 1d) [58]. Therefore, the gate dielectric consisted of a combination of air and SiO_2_. Since air has a relative dielectric constant (ε_r_ = 1) smaller than SiO_2_ (ε_r_ = 3.9), the threshold voltage increased. A further increase depended on the negative charges of CRP bound to the anti-CRP. Silicon-binding proteins (SBPs) linked to protein A (spA) were used to promote the attachment of anti-CRP onto the FET sensing surface. CRP was demonstrated to anchor only to anti-CRP, whereas no binding was reported to SBP-spA. When tested with human serum, this FET provided a LOD (0.1 ng/mL) similar to an ELISA kit (0.25 ng/mL), but covered a wider concentration range (FET up to 100 ng/mL, whereas ELISA up to 5 ng/mL). The authors aimed to prove that their sensor could be used to detect concentration changes with a resolution of about 1 µg/mL for the prevention of cardiovascular diseases; nevertheless, this sensor seems promising to detect the CRP concentration for monitoring wound healing.

Justino et al. fabricated a carbon nanotube FET (NTFET) [59] to exploit the large surface area and the sensitivity to organic molecules of carbon nanotubes (CNTs) for sensing applications [60,61]. In a back-gate configuration, the CNT channel was formed by casting 1.2 µL of single-wall CNTs (SWCNTs) dispersion on the FET. The anchoring of anti-CRP was performed without any adhesion-promoting layer because the SWCNTs directly bound to the amino group of anti-CRP through a non-covalent bond. The NTFET worked with CRP concentrations in the range 10^−4^–10^2^ µg/mL, which is broad enough to be used in several applications, such as the monitoring of cardiovascular diseases and wound inflammation/infection. The sensitivity corresponded to about a 0.2% decrease in the I_DS_ for an increase of 1% CRP level.

Lee et al. preferred a high electron mobility transistor (HEMT) where the channel was AlGaN/GaN to increase the sensitivity towards CRP [62]. Figure 1e shows a HEMT fabricated with a 2 μm thick resistive GaN layer, an 80 nm thick undoped GaN layer, and a 25 nm thick Al_0.3_Ga_0.7_N layer (30% Al). The gate length and width were 20 and 200 μm, respectively. The anti-CRP were anchored to a self-assembled monolayer (SAM) onto a Ni/Au gate. The selectivity was confirmed adding troponin T and observing no changes in the HEMT response. The HEMT was exposed to CRP concentrations from 0.01 ng/mL to 1000 ng/mL in PBS. Results showed a clear separation for concentrations greater than 10 ng/mL, whereas for smaller values the measurement noise was too high to discriminate between different CRP levels. Furthermore, the advantage of using an AlGaN/GaN is not highlighted as no comment on sensitivity was reported. However, the same research group proposed a new version of the HEMT with well-defined advantages such as an integrated quasi-reference electrode (QRE) to improve the device miniaturization and another HEMT with a Ni/Ti/Pt gate (thickness 20/20/100 nm) used as reference (ref-HEMT) to decrease the measurement noise in differential mode [63]. The LOD improved to 0.01 ng/mL.

A label-free electrolyte-gated organic FET (EGOFET) was described in [64]. In an EGOFET, the substrate is an organic semiconductor, whereas the interfaces between the gate electrode and the electrolyte and between the electrolyte and the organic semiconductor can be modified with the deposition of bioreceptors. Anti-CRP were directly adsorbed onto a poly-3-hexyl thiophene (P3HT, about 25 nm thick) layer and N-[tris(hydroxymethyl) methyl]acrylamide-lipoic acid conjugate (pTHMMAA) was used as blocking polymer. This sensor achieved a LOD of 220 ng/L and an upper detection limit of about 200 mg/L.

### 2.2. Optical Technologies

Optical technologies are widely used in biosensing and many clinical analyses are performed by means of optical tests, e.g., the ELISA assay. Literature reports several works where surface plasmon resonance (SPR), fluorescence, chemiluminescence, and other optical methods and devices (e.g., optical fibers and silicon photonics) are used to measure CRP levels. Optical measurements would be potentially useful for real time monitoring of CRP in wounds, but application in situ is often problematic due to the need of cumbersome readers or chemical labels.

#### 2.2.1. Surface Plasmon Resonance

SPR has been extensively used for the characterization of thin film and for the detection of biomolecules [65,66]. An SPR chip usually exploits an incident light through a prism coated with a gold layer in conditions of total internal reflection at the interface. At the point of reflection, an evanescent field penetrates the interface for a depth in the order of 1/4 of the incident light wavelength. In biosensing application, SPR measures the shift in the resonance angle after a change of the refractive index within about 300 nm of the sensor surface after the interaction with the molecules of interest. In 2006, Casa et al. used a basic SPR chip configuration where a layer of Cr/Au (5/33 nm thick) was sputtered onto the surface of a glass substrate (150 µm thick) [67]. The surface was activated with ester groups through several steps involving 4,4′-Dithiodibutyric acid (DDA), N-Hydroxysuccinimide (NHS) and 1-ethyl-3-3-dimethylaminopropylcarbodiimide hydrochloride (EDC) before anchoring the anti-CRP. In PBS, the LOD was 1 µg/mL, whereas the dynamic range extended up to 10 µg/mL. Although the dynamic range should be improved to monitor wound healing, this work paved the way towards the use of SPR to detect CRP concentration.

In the same year, Meyer et al. described another SPR immunosensor for CRP analysis [68]. A gold surface was first treated with APTES to form amino-groups and attach a biotin layer by means of NHS. The biotin layer was coated with streptavidin to promote the adhesion of biotinylated anti-CRP C6. A sandwich configuration was adopted where the biotinylated anti-CRP C6 (100 µg/mL) were used to capture CRP, the anti-CRP C2 (100 µg/mL) were used for detection, and BSA to block free binding sites. The range was 2–5 µg/mL, smaller than that reported by Casa et al. [67], but with a better LOD (2 µg/mL) and linearity (R^2^ = 0.98). The response time was less than 1 h.

In 2006, a third SPR chip for CRP was proposed for detecting both forms of CRP, i.e., the native pentameric CRP (pCRP) and the modified CRP (mCRP) [69]. A SAM of 16-mercaptohexadecanoic acid (MHA) was formed onto the gold surface and reacted with a solution of EDC/NHS. This activated SAM was then used to anchor a monolayer of protein G, which provided a high number of anti-CRP binding sites available on the protein. This immunosensor was tested with anti-CRP C8, which can bind to both pCRP and mCRP, and a non-linear response was obtained between 1 and 26 µg/mL. Specific anti-CRP for pCRP and mCRP were also used, 8D8 and 9C9 respectively, but more experiments were needed to improve the sensitivity of the biosensor.

In order to improve the density of binding sites and decrease non-specific bindings to the gold surface, Jung et al. proposed a layer of amide-linked (AL) NHS-dextran to anchor anti-CRP [70]. However, the performances of this approach were unsatisfactory in human serum probably because of different binding conditions at this specific pH level. In PBS, the SPR chip non-linearly responded from about 0.1 to 50 µg/mL.

Bini et al. covalently bound streptavidin to a carboxylated dextran-coated chip and then anchored an RNA aptamer to streptavidin via biotin binding [71]. This was probably the first attempt to bind CRP with an aptamer instead of anti-CRP. The best immobilization was achieved with an aptamer having a triethylene glycol (TEG) tail. With an aptamer concentration of 0.1 µM, the response of the SPR chip in a buffer solution at pH 6.5 was linear (R^2^ = 0.99) in the range 0.01–0.5 ppm with a LOD of 0.015 ppm. The authors observed that the presence of calcium ions (2 mM) improved the LOD and the maximum concentration to 0.005 ppm and 0.1 ppm, respectively. These results depended on the fact that calcium improves the stability of the pentameric form of CRP that circulates in human blood and avoids the formation of the mCRP form.

**Figure 2:**
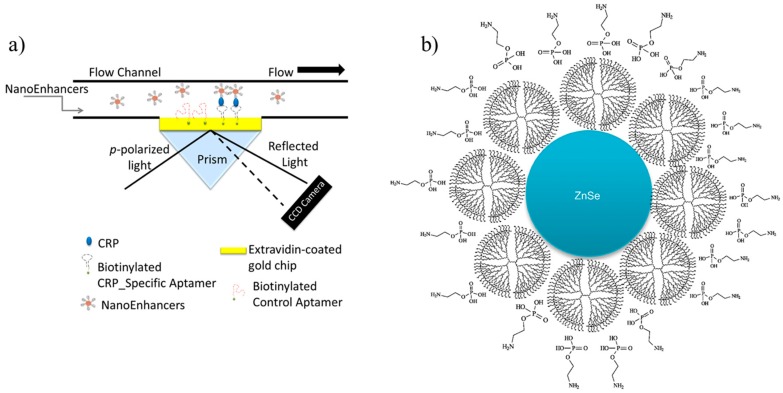
(**a**) Schematic view of the SPR chip with biotinylated aptamers coated by NIR-QDs (nanoenhancers) ([72], licensed under CC BY 3.0); (**b**) schematic view of the S-DAB–ZnSe–PEA QDs nanocomposite (Reprinted with permission from [86]).

Vance et al. proposed an SPR-based nano-aptasensor with a LOD of 5 fg/mL in spiked human serum [72]. A cystamine/glutaraldehyde/extravidin surface chemistry was used to bind biotin-labeled aptamers (59-GGGCCTCCGGT -TCATGCCGC-39), specific for CRP, onto a gold surface of a prism. The signal output was amplified (about 18%) by coating the aptamers with near-infrared quantum nanodots (NIR-QDs, nanoenhancers) (Figure 2a), thus obtaining a linear output in the range 5–5000 fg/mL. Aptamers to detect CRP were also used in [73]. The best aptamers were of the type CRP-40-17-x and CRP-80-17-x biotin, x = 3′ or 5′, and responded in the range 0.35–12.5 nmol/L.

Instead of anti-CRP or aptamers, Matsuura et al. used poly(2-methacryloyloxyethyl phosphorylcholine) (PMPC) to bind CRP onto the SPR chip, however the measurement was based on the binding of an anti-CRP/Cy5–streptavidin system to the PMPC CRP group [74]. Streptavidin and BSA reduced non-specific binding and improved the response. In 100-times diluted human serum, the SPR chip detected from 10 pM to 1000 pM of CRP with a good linearity (R^2^ = 0.98).

Wu et al. immobilized a biotinylated aptamer (6th-62-40-3′) onto a gold surface to bind CRP and used gold nanoparticles labeled with anti-CRP [75]. The selectivity of the system was verified with five proteins, namely human immunoglobulin G (IgG), human serum albumin (HSA), hemoglobin (Hb), transferrin (TRF) and myoglobin (Myo). This chip detected CRP from 10 pM to 100 nM in 100-times diluted human serum.

Choi et al. coated the surface of the SPR chip with a parylene N film, which could be used to immobilize CRP through physical adsorption [76]. Plasma treatment for 1 min at 100 W was used to introduce functional groups such as hydroxyl, peroxide, and carboxylic acids. Anti-CRP was anchored to a parylene N film and BSA was used to block non-specific binding. The SPR chip detected CRP concentrations from 1 ng/mL to 1 µg/mL, but more tests were needed to verify the repeatability and sensitivity.

A wide range of detection up to 70 mg/L with a LOD of 9 µg/L was achieved with a SPR chip combined with an optical fiber [77]. A 1 mm optical fiber with a polymethyl methacrylate (PMMA) core and a fluorinated polymer cladding was polished to expose the core. The exposed core was coated with photoresist and then a gold layer was sputtered onto it. The gold layer was functionalized with 11-mercaptoundecanoic acid in a H_2_O/ethanol solution (10% ethanol). The activation was performed with EDC/NHS to anchor covalently the anti-CRP, whereas BSA was used to block non-specific bindings. This biosensor worked in a thermo-stabilized microfluidic system and showed a good sensitivity (10^4^ nm/RIU, RIU = refractive index unit), with performances suitable for clinical use.

#### 2.2.2. Total Internal Reflection and Bragg Grating

Optical fibers exploit the total internal refraction (TIR) of light at the interface between a cladding (refractive indexes n_1_) and a core (n_2_) of the fiber when n_2_ > n_1_. TIR happens when the angle of incidence of light is greater than the critical angle θ_c_, as defined by the Snell law as $\theta_{c}=\sin^{-1}\frac{n_{1}}{n_{2}}$. A small fraction of light, i.e., the evanescent wave, penetrates the cladding for a fraction of wavelength (about 100 or 200 nm). If a sensing layer replaces part or all the cladding, the optical fiber becomes a sensor whose response is the change of the evanescent wave that interacts with this sensing layer.

Chou et al. immobilized anti-CRP onto the uncladded surface of an optical fiber in PBS and used BSA to block non-specific bindings [78]. This biosensor had a detection range between 5 and 12.5 µg/mL with a fluorescence response time of 10 min for both pCRP and mCRP.

Sridevi et al. modified an optical fiber to make a fiber Bragg grating (FBG), i.e., a fiber with a periodic refractive index of the core, thus filtering specific wavelengths [79]. The functionalization of the FBG was performed with graphene oxide where anti-CRP were immobilized. Graphene oxide was chosen because of the efficient binding to the hydrophilic surface of the fiber. The FBG detected CRP in the range 0.01–100 mg/L with a LOD of 0.01 mg/L. The measurement was not affected from interfering agents such as urea, creatinine and glucose ranging from 1 mg/L to 10 g/L.

#### 2.2.3. Fluorescence

In 2003, Wolf et al. developed a microfluidic chip including a fluorescent immunoassay to measure CRP in blood samples in the range 0.03 and 1 µg/mL [80]. The anti-CRP was labeled with a Cy3 dye and three minutes were needed for the fluorescence to reach 80% of the saturation signal. Although authors claimed that higher concentrations could be detected upon a dilution, this range is probably too narrow for wound healing, but sufficient for cardiovascular diseases.

Christodoulides et al. proposed a fluorescent microchip to measure the CRP concentration in saliva [81]. Saliva collection is easy and non-invasive, and for this reason can be used for monitoring patients over long periods of time [82,83,84]. In a sandwich detection approach, porous agarose microspheres (diameter about 280 µm) coupled with anti-CRP were used for capture CRP, whereas alexafluor-488 labeled anti-CRP and horseradish peroxidase (HRP) conjugated anti-CRP were used for fluorescence and colorimetric detection, respectively. The microspheres were in a micro-etched array of wells on a silicon wafer. The measurement took 12 min and the fluorescent assay was more sensitive than the colorimetric detection. The LOD was 5 fg/mL and the range was 10 fg/mL–10 pg/mL. Interestingly, only two out of thirty-six subjects had CRP concentrations detectable in saliva with ELISA assays, whereas the fluorescent microchip succeeded in all the patients.

Baldini et al. presented a micro-machined PMMA chip where the excitation radiation was perpendicular to the flow [85]. The chip had a large plastic optical fiber (diameter 1 mm) coupled with a waveguide fabricated in the PMMA cover that collected the fluorescence travelling anisotropically through the chip. Eudragit L1000 was used to form carboxylic groups on the PMMA surface that were activated with EDC/NHS to bind C5-clone capture antibodies. The binding of CRP was quantified with DY647-labeled C7-clone anti-CRP (ELISA sandwich). This chip covered the range 0.1–50 mg/L with a LOD of 0.004 mg/L.

Algarra et al. proposed an S-DAB–ZnSe–PEA QDs nanocomposite (Figure 2b), where S-DAB is a thiol polypropylenimine dendrimer (generation 5) and PEA is *O*-phosphorylethanolamine [86]. The excitation wavelength was 180 nm and the fluorescence spectrum spanned from 300 to 740 nm. The tests in human serum showed that lowering pH from 7 to 3 changed the shape of the fluorescence spectrum, with a decrease of the band at 465 nm and an increase at 391 nm. The opposite was observed when increasing pH up to 12, a behaviour that can be explained by the donor ability of hydrogen ions and the deprotonation of the amino groups. The effect of ionic strength was studied by adding KCl from 0.002 to 1.5 M. The fluorescence intensity decreased when the KCl concentration increased, probably because of a lower number of available binding sites. The authors claimed a linear trend in the fluorescence intensity for a CRP concentration from 0.5 to 10 mg/L. However, the high variability of fluorescence intensity limited the resolution of CRP concentration to more than 2 mg/mL.

#### 2.2.4. Chemiluminescence

A chemiluminescent sensor exploits the measurement of light intensity upon an emission due to a chemical reaction. Wang et al. used biotinylated CdSe/ZnS/PEG-COOH QDs, coated with avidin, to anchor anti-CRP [87]. This compound aggregated when mixed with streptavidin-coated superparamagnetic polystyrene beads (MB). The electro-chemiluminescence (ECL) was tested on gold-coated polycarbonate electrodes in PBS buffer (pH 7.5) for a CRP concentration of 10 µg/mL. This sensor proved suitable for measuring the CRP concentration over a range of 1–10 µg/mL.

Lee et al. fabricated a microfluidic chip integrating micropumps, microvalves, microinjector and vortex-type micromixer [88]. This chip automated the entire measurement process with 5 µL of human serum or blood samples in about 30 min using streptavidin-coated magnetic beads conjugated with biotin-labeled single-stranded DNA (ssDNA). The sequence of the synthesized ssDNA was 5′-GGCAGGAAGACAAACACGATGGGGGGGTATGATTTGATGTGGTTGTTGCATGATCGTGGTCTGTGGTGCTGT-3′. The LOD was 0.0125 mg/L and the Bland-Altman test showed that the data obtained using this chip were comparable with a commercial instrument to measure CRP levels.

### 2.3. Electrochemistry

Electrochemical methods for biosensing, e.g., potentiometry, amperometry and impedance spectroscopy, are widely used starting from the second decade on last century. These methods are usually easy to implement, suitable for miniaturization and with high sensitivity [89].

#### 2.3.1. Potentiometry

Zhu et al. presented a 3-electrode configuration with a saturated calomel reference electrode (RE), a platinum wire counter electrode (CE) and a gold working electrode (WE) [90]. The surface of the WE was functionalized with anti-CRP bound to gold nanoparticles, which were immobilized on a cysteamine monolayer. The cysteamine monolayer was used to increase the electron transfer distance, i.e., when CRP bound to the antibodies, the impedance increased. This immunosensor detected CRP concentration in the range 5–25 µg/mL, but repeatability and selectivity tests were not available.

A potentiometric analysis with ZnO nanotubes (WE) and Ag/AgCl as RE was described in [91]. ZnO nanostructures are biocompatible and have useful electric characteristics for biosensing, such as a high isoelectric point (9.5), which promotes the binding with proteins, and a high electron transfer. Anti-CRP were immobilized onto the ZnO surfaces by physical adsorption in PBS. Authors claimed a response time of 10 s with a detection range of 10^−6^–1 mg/L, however the error bars suggested a range of 10^−5^–^−1^ mg/L. This immunosensor had the best performances for pH ≤ 7 and T ≤ 55 °C, with the optimum conditions at the upper bounds.

#### 2.3.2. Amperometry

**Figure 3:**
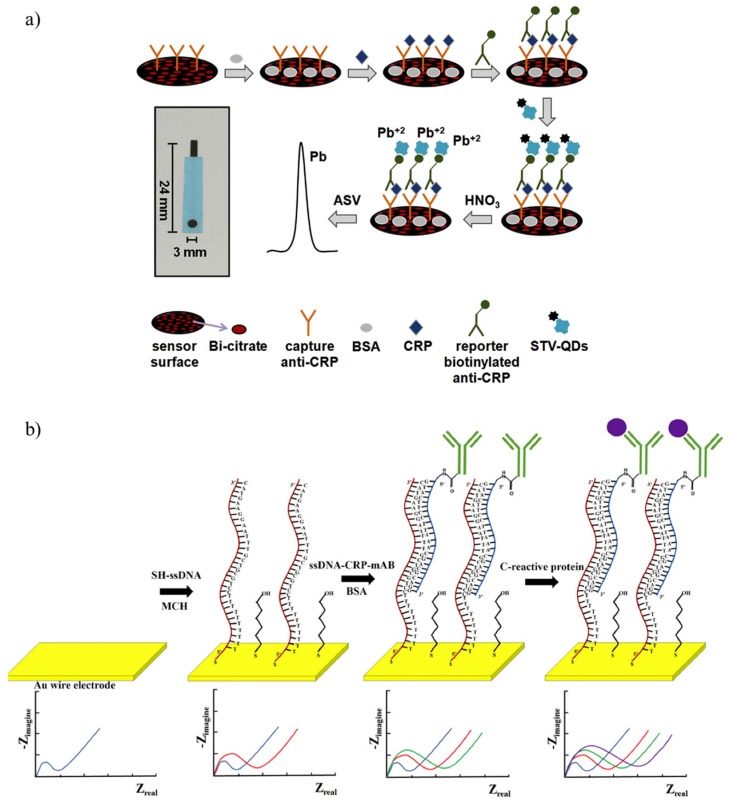
(**a**) Schematic process of the fabrication and working principle of the bismuth citrate-modified sandwich-type immunosensor proposed in (Reprinted with permission from [93]). The detection was performed by anodic scan voltammetry to detect the release of Pb(II) from the QDs in HNO_3_; (**b**) top: DNA-direct immobilization of anti-CRP onto a gold nanowire; bottom: changes in the electrochemical impedance after each step ([97], licensed under CC BY-NC-ND 4.0).

Fakanya et al. performed a chronoamperometric measurement with screen-printed gold electrodes (gold WE, carbon CE and Ag/AgCl RE) on polyethylene terephthalate (PET) [92]. Authors claimed that the covalent binding methods for anti-CRP were not demonstrated to be better than passive adsorption. Therefore, anti-CRP were passively adsorbed onto the WEs and tested in serum samples diluted 200-times in PBS-Tween. This immunosensor had a LOD of 2.6 ng/mL, which was close to that of an ELISA assay (1.9 ng/mL), and the maximum detectable CRP concentration was 100 ng/mL. Similar results were obtained by Kokkinos et al. who presented a sandwich-type screen-printed immunosensor with an Ag/AgCl RE and a Pt wire CE [93]. The WE was a mixture of bismuth citrate and graphite ink onto a graphite layer. Capture anti-CRP were physisorbed onto the WE and BSA was used to block non-specific bindings, whereas the detection antibodies were anchored to streptavidin lead sulfide QDs (strep-PbS QDs). The detection was performed by anodic scan voltammetry in HNO_3_ to detect the release of Pb(II) from the QDs. (Figure 3a). The CRP detection range was up to 100 ng/mL with a LOD of 0.05 ng/mL. Each immunosensor had a reproducibility of about 6% and showed comparable results to those of an ELISA assay.

The dynamic range was improved by Buch et al. using an Ag/AgCl RE and WE with a multi-wall CNT (MWCNT) modified carbon surface [94]. Human anti-CRP (50 mg/mL) conjugated with HRP were anchored to the protein A layer onto the WE. Protein A bound the anti-CRP so that the CRP recognition site kept its full activity and pointed towards the solution, thus improving the output signal. These electrodes were tested in PBS with different CRP concentrations by means of an amperometric measurement of the HRP enzyme when 3,5,3′,5′-tetramethylbenzidin (TMB) was added at the WE. The detection range covered 0.5–200 ng/mL with a response time of 10 min. The selectivity was confirmed testing the electrodes with the interfering agents, i.e., cholesterol, human IgG, triglycerides, and hemoglobin.

A wider dynamic range was obtained by Esteban-Fernández de Ávila et al. using screen-printed electrodes with gold WE and CE, and a pseudo Ag RE [95]. A layer of HOOC-modified magnetic beads (HOOC-MBs) was magnetically captured on the surface of the WEs. The HOOC-MBs were activated using EDC/NHS and covalently bound with biotinylated anti-CRP (1 µg/mL). Non-specific bindings were blocked adding an ethanolamine solution. The detection anti-CRP were labeled with a streptavidin-HRP conjugated compound (strep-HRP, dilution 1:1000) so that the target CRP was sandwiched between the biotinylated anti-CRP and the strep-HRP labeled anti-CRP. This magneto-immunosensor had a linear output range in 0.07–1000 ng/mL with a LOD of 0.021 ng/mL. The selectivity was confirmed adding solutions containing BSA, D-dimer, heparin, amino-terminal pro-B-type natri-uretic peptide (NT-proBNP) and cardiac troponin T, which did not interfere with the CRP detection.

#### 2.3.3. Electrochemical Impedance Spectroscopy

Electrochemical impedance spectroscopy (EIS) is the measurement of the electrical impedance of an interface in alternating current (AC) steady state with a constant direct current (DC) bias [96]. EIS can monitor the changes in the capacitance or in the charge transfer resistance (R_CT_) of the electrode-solution interface when molecules bind to the sensing surface. EIS is typically performed applying a small sinusoidal voltage at one or several frequencies and measuring the voltage/current ratio, i.e., the electrochemical impedance.

Songjaroen et al. described a label-free biosensor based on DNA-directed immobilization for detecting CRP [97]. The quality of the electrodes is critical for the performances of an electrochemical DNA assay [98]. The biosensor consisted of three 25-µm Au nanowires (the WEs) and one common 25-µm Ag nanowire as RE onto a PDMS support. Each WE was modified using a complementary strand of ssDNA conjugated to monoclonal anti-CRP (ssDNA-anti-CRP). Thiol groups promoted the binding of the ssDNA to the nanowires, whereas amine groups were used to bind anti-CRP to ssDNA (Figure 3b). The sensor calibration was performed in human serum and the CRP concentration was evaluated vs. R_CT_. The results showed that CRP concentration could be discriminated between about 12 and 25 mg/L, but sensitivity and reproducibility needed further investigation.

Nanoelectrodes (base diameter = 100 nm) were also proposed in [99]. These nanoelectrodes consisted of vertically aligned freestanding carbon nanofibers (VACNFs). The CE was a Pt wire and the RE a saturated calomel electrode. Anti-CRP were anchored to the nanoelectrodes after a pre-immersion in a HNO_3_ solution to form carboxylic groups and the activation with EDC and sulfo-NHS solutions in PBS. The LOD was about 11 ng/mL, however this sensor should be tested in biological samples.

### 2.4. Piezoelectricity

Piezoelectricity is the property of some materials to generate an electric field under mechanical deformation (direct piezoelectric effect) and to deform under the action of an electric field (reverse piezoelectric effect). Some examples of piezoelectric materials are crystals (e.g., SiO_2_, LiNbO_3_ and lead zirconate titanate (PZT)), polymers (e.g., polyvinylidene fluoride (PVDF)), semiconductors (e.g., ZnO and GaN), and biological molecules (e.g., DNA) [100,101]. In biosensing, quartz crystal microbalances (QCMs) exploit the change in the resonant frequency of quartz crystals upon a variation of the electrode mass due to the interaction with biomolecules. Therefore, if the crystal surface is functionalized, QCMs can measure the affinity of molecules as they bind to the crystal [102]. Similarly, changes in mass or viscoelasticity can be measured by frequency shifts when molecules bind to the surface of piezoresistive cantilevers [103].

#### 2.4.1. Quartz Crystal Microbalances

**Figure 4:**
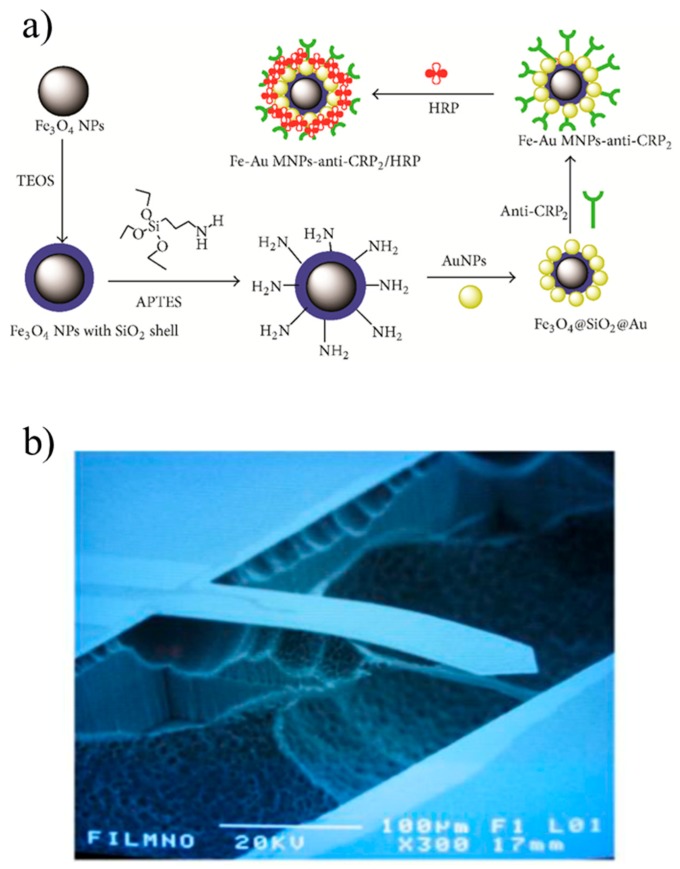
(**a**) Preparation of the Fe_3_O_4_-Au magnetic nanoparticles coated with anti-CRP and HRP ([104], licensed under CC BY 3.0); (**b**) SEM image of the microcantilever fabricated in [111] (licensed under CC BY 3.0).

Gan et al. functionalized the surface of a QCM with a combination of Fe_3_O_4_ and Au nanoparticles [104]. The Fe_3_O_4_ magnetic nanoparticles were coated with SiO_2_, immersed in APTES to form amine groups (NH_2_), and then coated with Au nanoparticles that were used to immobilize anti-CRP and HRP. HRP was used to catalyze 3,3′-Diaminobenzidine (DAB), a compound that in presence of H_2_O_2_ becomes insoluble and damp the QCM resonant frequency, thus amplifying the output signal (Figure 4a). At 37 °C and after 30 min, the CRP detection range was 0.003–200 ng/mL with a LOD of 1 pg/mL. This QCM was tested with CEA, human IgG and PSA, but these molecules did not provide any interfering signal. Gold nanoparticles were also used in a QCM working at higher concentrations [105]. The QCM surface was coated with capture anti-CRP, whereas the detection anti-CRP were anchored to gold nanoparticles. This QCM immunosensor had a linear response in 0.02–30 µg/mL in PBS and a sensitivity of about 11 Hz/µg/mL. The regeneration of the QCM immunosensor was obtained with a solution of 3 mol/L urea in PBS. The differences between tests in human serum and PBS were less than 10%, thus confirming the potential of the QCM immunosensor for detecting CRP levels in real samples.

Aizawa et al. proposed a method that did not need the fabrication of a sensing layer onto the QCM surface [106]. The authors immobilized anti-CRP on latex beads that, after the capture of the antigen, agglutinated and deposited on the QCM surface. This system responded up to about 670 µg/L, but the resolution was low.

Kim et al. used a QCM with an indirect competitive approach [107]. After a cleaning step, sulfo-succinimidyl 6-[3-(2-pyridyldithio)-propionamido]hexanoate (sulfo-LC-SPDP) was used to immobilize CRP (2 mg/mL) onto a gold electrode. After, a 200-µL mixture of anti-CRP (0.25 mg/mL) and CRP was added into the QCM cell with the CRP concentration ranging from about 0.13 to 25 ng/mL. Before each measurement cycle, 10 mM NaOH were used to regenerate the sensor surface and the LOD was 0.13 ng/mL. In order to improve the LOD (0.1 pM), the same authors published another work where the CRP on the surface, the free CRP and streptavidin-coated gold nanoparticles competed to bind with biotinylated anti-CRP [108].

#### 2.4.2. Microcantilever

Lee et al. fabricated a microcantilever consisting of SiO_2_/Ta/Pt/PZT/Pt/SiO_2_ on silicon nitride (total thickness = 3.5 µm) [109]. The microcantilever was coated with Cr/Au and then a Calixcrown (Calixarene derivative) SAM was formed to anchor anti-CRP. CRP was bound with a Cy3 dye and fluorescence measurements confirmed that CRP only interacted with anti-CRP. Tests showed that this microcantilever could achieve a sub-nanomolar sensitivity with a response time of 60 min.

Wee et al. exploited the piezoresistive properties of doped silicon [110]. The microcantilever consisted of a multilayer of Si_3_N_4_/poly-Si/SiO_2_/Si_3_N_4_ where the boron-doped poly-Si was the piezoresistive element. The use of silicon nitride was two-fold: the bottom Si_3_N_4_ layer was a structural layer, whereas the upper Si_3_N_4_ layer was used to passivate and insulate the microcantilever. A layer of Cr/Au was evaporated onto the microcantilever for immobilizing anti-CRP, whereas the bottom of the microcantilever was not biologically active. The microcantilever was tested at 100 ng/mL and 1 µg/mL CRP. Unfortunately, at the highest concentration, the response fluctuated without stabilizing.

Yen et al. described a SiO_2_/Si_3_N_4_/doped poly-Si/Si_3_N_4_/ SiO_2_ microcantilever, where silicon nitride was used for passivation and insulation, whereas the oxide to balance the built-in stress (Figure 4b) [111]. This microcantilever had a linear response in the range 10–100 µg/mL (detection time about 50 min).

Table 1 summarizes the main characteristics of the biosensors for measuring CRP concentration.

## 3. Sensors for Temperature and pH

The measurement of the wound temperature and pH in clinical practice are usually performed by trained clinical personnel with cumbersome instruments such as thermographic cameras and pH meters. However, these instruments are not suitable for a real-time monitoring of the wound. The measurement of pH can also be performed with litmus paper, but this method has a low accuracy, is single use, and depends on the user’s decision to determine the color of the paper. These limitations hinder the continuous clinical evaluation of the status outside clinical settings or specialized labs. The clinicians are not aware of the efficacy of the wound treatment when the patient is at home because the constant monitoring of objective biomarkers and predictors are not available. Conversely, the possibility to follow the evolution of the wound status even when the patient is not hospitalized would allow a better and personalized therapy to be administered and a prompt intervention in case of infection. Two recent reviews described the advances in the development of temperature and pH sensors for wound monitoring [1,112]. However, in the last couple of years, literature has reported several temperature and pH sensors that could be used for monitoring wound status.

McNeill et al. proposed a flexible and wearable sensor for the local monitoring of skin pressure, relative humidity and temperature [113]. The substrate was a flexible polyimide printed circuit board on an adhesive film (OPSITE by Smith & Nephew), whereas the temperature sensor was a SHT3x-ARP (accuracy ±0.3 °C, Sensirion). This approach can be used to monitor the perilesional skin, but is not suitable for the wound bed. A chip-free flexible patch was reported by Yamamoto et al. and had a temperature sensor on a PET film [114]. The bottom side of the film was coated with a layer of ethoxylated polyethylenimine (PEIE) and MWCNTs mixed with polydimethylsiloxane (PDMS) with a 3 wt % PEIE concentration in PDMS with 10 wt % MWCNTs. The temperature sensor was resistive and consisted of a printed layer of poly(3,4-ethylenedioxythiophene)polystyrene (PEDOT:PSS). The sensing mechanism depended on the electron hopping through the PET film between MWCNTs and PEDOT:PSS. The best results were obtained with a 25 µm thick film with a sensitivity of about 0.85% ΔR/°C (ΔR is the resistance variation).

Nakata et al. fabricated a wearable and flexible patch on PET for monitoring skin temperature [115]. The temperature sensor consisted of a solution of a MWCNTs ink and 1.3 wt % PEDOT:PSS in water (ratio 1:3) that was printed and cured at 70 °C. This sensor was tested between 22 and 36.5 °C and the sensitivity, defined as the relative variation of resistance with respect to the resistance at room temperature, was 0.85%/°C, whereas the repeatability was not reported.

Trung et al. developed an all-elastomeric transparent stretchable-gated temperature sensor [116]. This FET had source, drain and gate electrodes made of a PEDOT:PSS and polyurethane (PU) composite. The gate dielectric was made of PU, whereas the temperature-sensitive FET channel was a nanocomposite of reduced graphene oxide and PU. The sensor could be worn on the skin and was fully functional for a strain up to 70% and after 10,000 cycles of stretching at 30% strain. The working range was 30–80 °C, whereas the sensitivity was about 1.34% resistance change per °C that yielded to detect changes of 0.2 °C.

Literature reports other sensors for monitoring the temperature of the perilesional skin [117,118,119], whereas only a few studies proposed temperature sensors for wound bed. A sensor in direct contact with the wound bed would pave the way towards a continuous real time monitoring of wound status.

**Figure 5:**
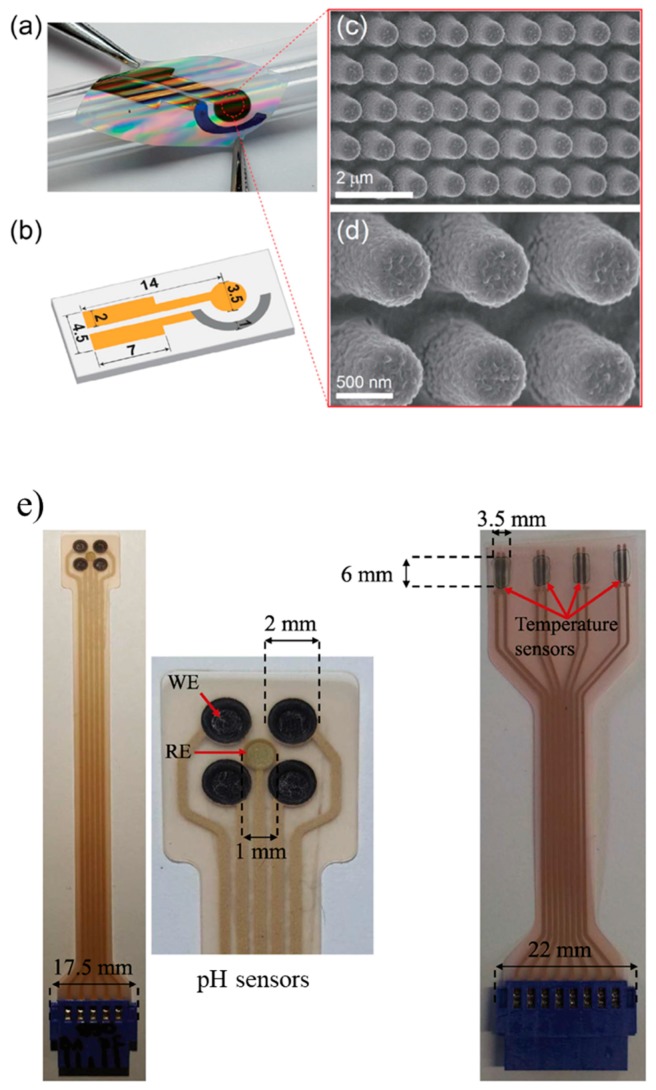
(**a**) An example of a flexible PAN pH sensor with (**b**) sensor dimensions; (**c**,**d**) SEM images of the PAN sensor (Reprinted with permission from [128]); (**e**) examples of the pH (left) and temperature (right) screen-printed sensors to use in direct contact with a wound (Reprinted with permission from [120]).

Salvo et al. explored the possibility to measure wound temperature and pH using reduced graphene oxide (rGO) and graphene oxide (GO), respectively [120,121,122]. Both sensors were fabricated onto a flexible PET substrate (125 µm thick) and were capable of measuring four pH and four temperature values (Figure 5e). The temperature sensor was resistive, whereas the pH sensor exploited the protonation/deprotonation of the hydroxyl (OH) and carboxyl (COOH) groups of a GO layer, and the reversible structural change of the epoxy groups (COC) on the basal plane of a GO layer at different pH [123]. The pH cell consisted of an Ag/AgCl RE and four WEs. The biocompatibility was assessed in a broth medium that included minimum essential medium (MEM) with 10% FBS and MRC-5 (human fibroblast cell line, p33) cells. In human serum between 25 and 43 °C, the temperature sensor had a sensitivity of 110 ± 10 Ω/°C, repeatability of ±0.25 °C and an error of 0.4 ± 0.1 °C compared with a gold standard. In buffer solutions, the pH sensor had a sensitivity of 40 ± 4 mV/pH in the pH range 4–10 with a repeatability error of ± 4 mV and a hysteresis of 0.15 pH units. The pH sensor was also tested in human serum over 1 week and in human plasma over 1 month, and had an acceptable difference of about 0.1 ± 0.1 pH units compared to a commercial pH meter. In the range 25–45 °C, the temperature dependence was 0.01 pH/°C.

In 2016 and 2017, two research groups presented two pH sensors for in vivo applications, but these devices seem too invasive for continuous wound monitoring on human subjects [124,125]. Rahimi et al. fabricated a pH sensor with a specific application for wound assessment [126]. A 3 × 3 flexible array of pH sensors was fabricated onto a paper substrate and embedded into a wound dressing. Each sensor consisted of an Ag/AgCl RE and a carbon WE coated with PANI. The PANI layer was doped with HCl vapor to increase the conductivity. The pH sensor was tested in buffer solutions and had a sensitivity of about −58 mV/pH in the pH range 2–10 and good linearity (R^2^ = 0.95). The linearity improved in the pH range 4–10 where R^2^ = 0.97, but the sensitivity decreased to about −50 mV/pH. After 24 h, the drift was about 0.01 pH/h. The response time was about 12 s for pH increasing from 6 to 8, whereas about 36 s from pH 8 to 6. The biocompatibility was confirmed testing the pH sensor over seven days with human keratinocytes cultured in Dulbecco’s Modified Eagle Media (DMEM) with 10% fetal bovine serum (FBS) and 1% penicillin–streptomycin.

Panzarasa et al. used a pyranine-benzalkonium ion pair incorporated in membranes and wound dressings to detect pH changes in the range 5.5–7.5, however the standard deviation was too high to discriminate pH values that differs for less of about 0.5 pH units [127].

A potential pH sensor that could integrated into a dressing was described by Yoon et al. [128]. A matrix of nanopillars (mixture of polyurethane acrylate and NOA63, diameter about 400 nm) was fabricated on a PET substrate. The WE and RE were fabricated starting with Au/Ti and Ag/Ti depositions, respectively. An Ag/AgCl RE was obtained by electroplating chloride on silver and the coated with the dielectric ink ESL 242-SB mixed with KCl (30 wt %). The WE was obtained by the electrochemical deposition of polyaniline (PANI, about 50 nm thick) on a gold surface (Figure 5a–d). This pH sensor was tested in buffer solution from pH 2 to 12. The response was linear all over the tested range (R^2^ = 0.99) and the sensitivity was 60.3 mV/pH, even if bent for 1000 cycles. The response time was less than 1 s. Authors verified that the response time increased to about 9 s without the nanopillars structure. The repeatability tests showed a negligible hysteresis, whereas the drift evaluated from 5 to 12 h upon immersion in a buffer solution was 0.64 mV/h at pH 5 and 0.49 mV/h at pH 7. In presence of interfering ions such as Na^+^, K^+^, NH^4+^, Ca^2+^, and Mg^2+^, the pH sensor showed an excellent selectivity. This pH sensor obtained comparable results to those of a commercial pH meter when tested with real samples such as coke, coffee, water, and orange juice.

An optical fiber could be a promising option for the real time wound monitoring, but fabrication and embedding into a dressing or a bandage does not seem straightforward because of movement and bending noise. An optical fiber could also be inserted into the drainage tube used to draw exudate during negative pressure therapy. However, the flowing time of exudate through the tube should be taken into account as a long transit time could alter the pH value [129]. A promising work was a miniature Fabry–Perot interferometer used to monitor pH values from 4.3 to 6.9 with a sensitivity of 11 nm/pH [130]. The sensitive layer was a polyvinyl alcohol/polyacrylic acid (PVA/PAA) hydrogel, whereas the interferometer consisted of a hollow-core photonic crystal fiber (HCPCF) and single mode fiber (SMF).

## 4. Conclusions

The literature reports a large number of biosensors that measure CRP trying to achieve similar or better performances than ELISA assays. Most of the research efforts involving CRP aim at cardiovascular applications, but some of these biosensors would also be suitable for monitoring the wound status. CRP concentration <8 mg/L is considered a normal clinical condition in wounds [27], whereas higher values can be used to discriminate different critical conditions. Cerveró-Ferragut et al. reported that proliferative and inflammatory phases had an average CRP level equal to 90 mg/L, which dropped to 5.2 mg/L in the maturation phase [131]. An average CRP level of about 66 mg/L was reported for trauma-related chronic wounds [132], whereas wound infections following surgically managed fractures were associated with a CRP concentration of 141 mg/L [133]. In diabetic foot ulcers, a CRP concentration threshold of 17 mg/L can be used to distinguish between grade 1 (uninfected) and grade 2 (mild severity infection) ulcers [134], while in venous ulcers the infection threshold was set to 20 mg/L [135]. In the case of diabetic foot osteomyelitis, an average of 95.6 mg/L was found for patients who needed additional amputation, whereas healing and remission were found for concentrations <40 mg/L [136]. A cut-off level of 36 mg/L was reported for incisional surgical site infection [137]. These values show that many biosensors would be able to classify between healing and non-healing/infected wounds (Table 1). However, only few have a sufficiently wide range (up to 100 mg/L in [55,59,79] and up to 200 mg/L in [64,111]) that would allow the full spectrum of wound status to be covered. The FET-based biosensors seem to be the most promising devices for wound monitoring. Although some of them had a working range similar to that of ELISA, validation tests against commercial products are needed. Furthermore, besides increasing the detection range, more tests on real samples followed by sensitivity (often not reported or confused with LOD), reproducibility, drift, cross-interference and shelf life analyses should be performed before adopting these biosensors for practical use. The sample size and preparation was seldom discussed, thus the effects on the measured concentration and detailed protocols should be included in further works. Another breakthrough could be the integration of these biosensors into dressing or bandage for a continuous real time monitoring of wound status. Presently, there are no available devices and the sample dilution is unpractical for this kind of wearable sensors.

In the last few years, some temperature and pH sensors have paved the way for a continuous real time wound monitoring by means of wearable devices or materials suitable for integration in commercial dressings [120,121,122,126,127]. Nevertheless, tests on human patients are needed to confirm the reliability of these sensors for practical use.

## Figures and Tables

**Table 1:** Analytical characteristics for different CRP biosensors reported in the literature (N.A. not available).

Measurement Technique/Device	Detection Layer	Medium	Range	Measurement Time (Excluding Preparation)	Sensitivity	Reference
***FET***						
EGFET	anti-CRP	PBS	3–10 mg/L	N.A.	N.A.	[49]
FET	Cysteine-tagged protein G + anti-CRP	PBS	3–20 mg/L	≈10 min	≈ 0.01 µg/mL/(A/A)	[51]
SiNW-FET	anti-CRP	PBS	1 fM–1 nM	≈200 s	N.A.	[52]
SiNW-FET	anti-CRP	Serum	3.2–10.4 mg/L	N.A.	N.A.	[53]
SiNW-FET	Sol-gel + anti-CRP	Serum	0.12–10 µg/L	N.A.	N.A.	[54]
NG-SiNW-FET	anti-CRP	PBS	100 mg/L	N.A.	N.A.	[55]
SiNW-FET	anti-CRP	PBS	0.1–10 mg/L	<100 s	16–39% of conductance variation	[56]
MOSFET-BJT	anti-CRP	PBS	1 pmol/L–1 µmol/L	N.A.	0.80 µA/decade @ base current = −10 µA for BJT	[57]
NG-FET	SBP-spA + anti-CRP	Serum	100 µg/L	N.A.	3.4 V/g/mL	[58]
NTFET	anti-CRP	PBS	10^−4^–10^2^ mg/L	N.A.	0.2% of current variation/0.1% CRP concentration	[59]
Differential mode-HEMT	anti-CRP	PBS	0.01–1000 µg/L	≈100 s	N.A.	[63]
EGOFET	P3HT + anti-CRP	PBS	220 ng/L–200 mg/L	N.A.	N.A.	[64]
***Optical***						
SPR	anti-CRP	PBS	1–10 mg/L	10 min	N.A.	[67]
SPR	Biotinylated anti-CRP	PBS	2–5 mg/L	<1 h	~10.7 A.U./(µg/mL)	[68]
SPR	Protein G + anti-CRP	Tris buffered saline–calcium	1–26 mg/L	10 min	N.A.	[69]
SPR	amide-linked NHS-dextran + anti-CRP	Serum	0.1–50 mg/L	N.A.	N.A.	[70]
SPR	RNA aptamer	Serum	0.005–0.1 ppm	15 min	~2827 Resonance unit/ppm	[71]
SPR	Aptamer + QDs	Serum	5–5000 pg/L	~20 min	N.A.	[72]
SPR	DNA aptamers	N.A.	0.35–12.5 nmol/L	N.A.	0.0044 degree shift/nmol/L	[73]
SPR	PMPC	Serum	10–1000 pM	N.A.	N.A.	[74]
SPR	Aptamer	Serum	10 pM–100 nM	N.A.	N.A.	[75]
SPR	Parylene-N + anti-CRP	N.A.	1–1000 µg/L	~20 min	N.A.	[76]
SPR + optical fiber	anti-CRP	Serum	9 µg/L–70 mg/L	~10 min	10^4^ nm/RIU	[77]
Optical fiber	anti-CRP	PBS	5–12.5 mg/L	10 min	10^7^ s^−1^/molar for pCRP; ~317 × 10^3^ s^−1^/molar for mCRP	[78]
Fiber Bragg grating	GO + anti-CRP	Serum	0.01–100 mg/L	N.A.	N.A.	[79]
Fluorescence	anti-CRP	Plasma	0.03–5 mg/L	180 s	N.A.	[80]
Fluorescence	anti-CRP	Saliva	10 pg/L–10 ng/L	12 min	N.A.	[81]
Fluorescence	anti-CRP	HEPES	0.1–50 mg/L (LOD 0.004 mg/L)	~26 min	N.A.	[85]
Fluorescence	S-DAB–ZnSe–PEA QDs + anti-CRP	Serum	0.5–10 mg/L	N.A.	−2 × 10^−8^ A.U./log(mg/L)	[86]
Chemiluminescence	CdSe/ZnS/PEG-COOH QDs + anti-CRP	PBS	1–10 mg/L	>3 h	N.A.	[87]
Chemiluminescence	ssDNA	PBST	0.0125–10 mg/L	30 min	≈ 171/$\sqrt{Conc.\left(\frac{mg}{L}\right)}$	[88]
***Electrochemistry***						
Potentiometry	Cysteamine + Au NPs + anti-CRP	NaCl	5–25 mg/L	10 min	N.A.	[90]
Potentiometry	ZnO + anti-CRP	PBS	10^−6^–1 mg/L	<10 s	~13 V/log[Conc.(mg/L)]	[91]
Amperometry	anti-CRP	Serum	2.6–100 µg/L	200 s	0.026 µA/(ng/mL) in 2.6–50 ng/mL	[92]
Amperometry	anti-CRP + lead sulfide QDs	HNO_3_	0.2–100 µg/L, LOD 0.05 µg/L	>2 h	N.A.	[93]
Amperometry	anti-CRP + HRP + TMB	PBS	0.5–200 µg/L	10 min	N.A.	[94]
Amperometry	HOOC-MBs + anti-CRP	Serum	0.07–1000 µg/L, LOD 0.021 µg/L	90 min	2 × 10^−7^ A	[95]
EIS	ssDNA + anti-CRP	Serum	12–25 mg/L	>10 min	0.0067 Ω/(mg/L) in 3.125–25 mg/L	[97]
EIS	anti-CRP	PBS	~0.4–42 nM, LOD 11 µg/L	>1 h	N.A.	[99]
***Piezoelectricity***						
QCM	Fe_3_O_4_ and Au NPs + anti-CRP	PBS	0.003–200 µg/L, LOD 1 ng/L	30 min	N.A.	[104]
QCM	Au NPs + anti-CRP	PBS	0.02–30 mg/L	>1 h	11 Hz/(µg/mL)	[105]
QCM	anti-CRP	Serum	~170–667 µg/L	~60 min	~10 Hz/(µg/dL)	[106]
QCM	SA-coated Au NPs + anti-CRP	PBS	0.1 pM–0.53 nM	~25 min	N.A.	[108]
Microcantilever	anti-CRP	PBS	100 µg/L and 1 mg/L	>15 min	N.A.	[110]
Microcantilever	anti-CRP	PBS	1–200 mg/L, linear in 10–100 mg/L	~50 min	N.A.	[111]
